# Structural Brain Network Reorganization and Social Cognition Related to Adverse Perinatal Condition from Infancy to Early Adolescence

**DOI:** 10.3389/fnins.2016.00560

**Published:** 2016-12-08

**Authors:** Emma Muñoz-Moreno, Elda Fischi-Gomez, Dafnis Batalle, Cristina Borradori-Tolsa, Elisenda Eixarch, Jean-Philippe Thiran, Eduard Gratacós, Petra S. Hüppi

**Affiliations:** ^1^Fetal i+D, Fetal Medicine Research Center, Barcelona Center for Maternal-Fetal and Neonatal Medicine (Hospital Clínic and Hospital Sant Joan de Deu), Institut d'Investigacions Biomèdiques August Pi I Sunyer, University of BarcelonaBarcelona, Spain; ^2^Experimental 7T MRI Unit, Institut d'Investigacions Biomèdiques August Pi I SunyerBarcelona, Spain; ^3^Signal Processing Laboratory 5, École Polytechnique Fédérale de LausanneLausanne, Switzerland; ^4^Division of Development and Growth. Department of Pediatrics, University Hospital of GenevaGeneva, Switzerland; ^5^Centre for the Developing Brain, Division of Imaging Sciences and Biomedical Engineering, King's College LondonLondon, UK; ^6^Centre for Biomedical Research on Rare DiseasesBarcelona, Spain; ^7^Department of Radiology, University Hospital Center and University of LausanneLausanne, Switzerland

**Keywords:** connectome, intrauterine growth retardation, birth weight, executive function, neurodevelopment, preterm infants

## Abstract

Adverse conditions during fetal life have been associated to both structural and functional changes in neurodevelopment from the neonatal period to adolescence. In this study, connectomics was used to assess the evolution of brain networks from infancy to early adolescence. Brain network reorganization over time in subjects who had suffered adverse perinatal conditions is characterized and related to neurodevelopment and cognition. Three cohorts of prematurely born infants and children (between 28 and 35 weeks of gestational age), including individuals with a birth weight appropriated for gestational age and with intrauterine growth restriction (IUGR), were evaluated at 1, 6, and 10 years of age, respectively. A common developmental trajectory of brain networks was identified in both control and IUGR groups: network efficiencies of the fractional anisotropy (FA)-weighted and normalized connectomes increase with age, which can be related to maturation and myelination of fiber connections while the number of connections decreases, which can be associated to an axonal pruning process and reorganization. Comparing subjects with or without IUGR, a similar pattern of network differences between groups was observed in the three developmental stages, mainly characterized by IUGR group having reduced brain network efficiencies in binary and FA-weighted connectomes and increased efficiencies in the connectome normalized by its total connection strength (FA). Associations between brain networks and neurobehavioral impairments were also evaluated showing a relationship between different network metrics and specific social cognition-related scores, as well as a higher risk of inattention/hyperactivity and/or executive functional disorders in IUGR children.

## Introduction

Brain development consists of a genetically controlled but environmentally modulated complex process starting early in fetal life, which makes it very sensitive to adverse intrauterine environment and/or to a premature exposure to extrauterine medium. Alterations in fetal brain development can result in long-term structural brain reorganization as well as in social cognition impairments in childhood and adolescence (Leitner et al., 2000; Wiles et al., 2006; Baschat, 2011; Guellec et al., 2011). Namely, intrauterine growth restriction (IUGR), a condition affecting 5–10% of all pregnancies involving a decrease in the amount of nutrients and oxygen arriving to the fetus, has consequences on the developing brain associated to short- and long-term impairments in both brain function and structure (Rees et al., 2008; Levine et al., 2015).

In order to better understand the long-term consequences of adverse perinatal condition, the analysis throughout childhood of the changes in brain structure and neurobehavior associated to this condition becomes essential. This longterm perspective has been considered in some studies describing the neurobehavioral and cognitive dysfunctions associated with perinatal conditions from infancy to adolescence (Heinonen et al., 2010; Baschat, 2011; Guellec et al., 2011; Levine et al., 2015). For instance, a large French population based study has shown that preterm infants (29–32 weeks of gestation) born with IUGR have a very high incidence (40%) of cognitive delay at 6 years of age (Claas et al., 2011). Indeed, the neurodevelopmental outcome of high risk populations including impairment in cognitive, executive, motor and behavioral functions has been described to be present throughout childhood, with consequences observed even in adult life (Walker and Marlow, 2008; Østgård et al., 2014b), though with smaller incidence (Saigal et al., 2006; Saigal, 2014).

However, most studies describing structural changes have focused on a single time point, from fetal life (Egaña-Ugrinovic et al., 2013, 2014a,b; Sanz-Cortés et al., 2014, 2015) up to early adulthood (Eikenes et al., 2012; Østgård et al., 2014a), including neonates (Borradori Tolsa et al., 2004; Dubois et al., 2008), 1-year-old children (Padilla et al., 2011; Batalle et al., 2012, 2013) and school-age children (Fischi-Gómez et al., 2015). These studies, mainly based on magnetic resonance imaging (MRI), describe the brain structural alterations at different development stages as a consequence of IUGR. Some of them also analyze the association between structural alterations and functional neurodevelopmental outcome at different ages. For example, IUGR has been related to reductions in overall brain volumes, with regional changes in gray matter (GM) and white matter (WM) volumes (Padilla et al., 2011) and specifically in cortical GM volume (Borradori Tolsa et al., 2004; Egaña-Ugrinovic et al., 2014b), hippocampus (Lodygensky et al., 2008), corpus callosum and brain-stem and cerebellum ratio (Sanz-Cortés et al., 2014). Changes in cortical surface and folding (Dubois et al., 2008; Egaña-Ugrinovic et al., 2013; Østgård et al., 2014a) and differences in WM integrity (Eikenes et al., 2012) have been also described.

Furthermore, changes in brain connectivity in IUGR subjects have been identified using connectomic analysis, that allows to model the brain structural or functional connections as a network: the connectome. It can be mathematically described and analyzed using graph metrics (Rubinov and Sporns, 2010; Sporns, 2012), which help to understand its large-scale structural topology. Diffusion-MR based network analysis has been used to describe quantitative differences in global brain connectivity in IUGR and their association with impairments in the neurodevelopmental outcome (Batalle et al., 2012; Fischi-Gómez et al., 2015). Additionally, connectomic analysis has also been performed on a rabbit model of IUGR, showing changes in some network metrics and its association with functional outcome in pre-adolescent adult rabbits (Batalle et al., 2014).

Connectomics has been used to describe the evolution of brain networks with age, since it provides a set of quantitative measures describing global and local brain network organization, whose changes over time can be quantified. Brain development in healthy subjects has been assessed by means of connectomics in different age ranges (Hagmann et al., 2010; Fan et al., 2011; Yap et al., 2011; Dennis and Thompson, 2013; Tymofiyeva et al., 2013; Ball et al., 2014; Pandit et al., 2014; Koenis et al., 2015; van den Heuvel et al., 2015; Zhao et al., 2015). However, the developmental trajectory in subjects who had suffered a perinatal adverse condition, such as IUGR, has not yet been characterized.

In the present study, connectomics was used to analyze the development of brain networks in children that had suffered IUGR. From infancy to early adolescence, brain structure was quantified by network metrics at three different time points (1, 6, and 10 years of age), as a function of age and group. Because of the evidence of neurobehavioral impairment in these children, neurobehavioral scores were correlated with network measures to determine the relationship between brain structure and neurobehavioral outcome from infancy to childhood.

## Materials and methods

### Subjects

Three cohorts of prematurely born infants (gestational age (GA) at birth between 28 and 35 weeks) were considered in this study. Each cohort was analyzed at a different age:

- First cohort (C1) consisted of 15 subjects, recruited at Hospital Clínic de Barcelona. Infants underwent MRI acquisition at 1 year of age and neuropsychological evaluation at 2 years.
- Second cohort (C6) was composed by 18 subjects, recruited at the Hôpitaux Universitaires de Genève (HUG). At 6 years of age, both MR imaging and neurobehavioral evaluation was performed.
- Third cohort (C10) was composed by 16 subjects, recruited at the Hôpitaux Universitaires de Genève (HUG). Both MR imaging and neurobehavioral assessment was performed at 10 years of age.

In each cohort, children were classified into two groups: children born preterm with normal growth (controls) and children born preterm with IUGR. Group distribution in the three cohorts, average GA and the age at which the MR scan and the neuropsychological evaluation were performed are detailed in Table 1. IUGR was defined as estimated fetal weight below the 10th percentile according to local standards (Figueras et al., 2008) confirmed at birth and abnormal Doppler defined as: umbilical artery pulsatility index above 95th centile and/or cerebroplacental ratio below 5th centile and/or mean uterine artery pulsatility index above 95th centile.

**Table 1:** **Group distribution and basic information of the three cohorts in the study: gestational age (GA) in weeks at birth, age at MR-scan (years), age at neurobehavioral evaluation (years)**.

	**1-year-old cohort**		**6-year-old cohort**		**10-year-old cohort**	
	**Controls**	**IUGR**	**Controls**	**IUGR**	**Controls**	**IUGR**
Sample	7	8	8	10	8	8
GA	32.2±1.7	30.9±1.7	32.4±2.3	32.6±1.5	32.5±1.8	32.7±1.5
Age at MR-scan	1.1±0.2	1.0±0.1	6.8±0.6	6.9±0.7	10.0±1.0	10.2±1.0
Age at neuropsy. test	1.7±0.3	1.7±0.4	6.3±0.7	6.2±0.5	9.8±1.0	10.0.3±0.9

Perinatal data was prospectively recorded for all subjects, including birth weight (BW), GA at birth and gender. Pregnancies were dated according to the first-trimester crown-rump length measurements (Robinson and Fleming, 1975). Infants with chromosomal, genetic or structural defects, signs of intrauterine infection or neonatal early onset sepsis as defined by positive blood culture within the first 72 h of life, and significant neonatal morbidities were excluded from this study. All of the children considered in the study were free from medication and from psychiatric or neurological disease. Parental socio-economic status and maternal education were also recorded.

All studies were approved by the ethical committee of the corresponding hospital and written informed consent was obtained from the parents or legal guardians of all the participants. The same group inclusion criteria were applied in both hospitals.

### Image acquisition

MRI examinations were performed on 3T Siemens TrioTim systems (Siemens Medical Solutions, Erlangen, Germany). C1 cohort was scanned at Hospital Clínic de Barcelona and C6 and C10 were scanned at Hôpitaux Universitaires de Genève. For each subject, high-resolution T1-weighted (T1w) images were acquired using a 3D magnetization prepared rapid acquisition gradient echo (MPRAGE) sequence. Diffusion weighted images (DWI) were acquired using a diffusion sensitized single echo planar imaging (SE-EPI) sequence covering 30 diffusion directions (b_0_ = 1000 m/s^2^) and a baseline image without diffusion weight. Acquisition parameters are detailed in Table 2. No subject was sedated during the protocol. Acquisition at 1 year of age was performed during natural sleep (Padilla et al., 2012).

**Table 2:** **Magnetic resonance acquisition parameters for the three cohorts under study**.

	**Cohort**	**TR/TE (TI) (ms)**	**In-plane resolution (mm)**	**Slice thickness (mm)**
T1-w	*C1*	2050/2.14 (1050)	0.86 × 0.86	0.9
	*C6-C10*	2500/2.91 (1100)	1 × 1	2
DWI (30	*C1*	9300/94	1.64 × 1.64	3
directions)	*C6-C10*	1020/107	1.82 × 1.82	2

### Image processing: obtaining the structural connectome

The extraction of the whole brain structural connectivity matrices (connectomes) followed the methodology described in Batalle et al. (2012). In short, for each subject the structural brain connectivity was inferred by combining the information from brain parcellation and fiber tractography. The entire processing pipeline can be sequentially divided in the following steps.

#### Brain tissue segmentation

For each subject, the T1w image was segmented into WM, GM and cerebrospinal fluid (CSF) using the unified segmentation model from SPM package (Ashburner and Friston, 2005), which, based on a priori maps, describes the probability of having a given kind of tissue in a given brain position. By default, SPM provides probability maps derived from adult brains, suitable for C6 and C10 cohorts, but not appropriated for the youngest cohort, where specific probability maps adapted to 1-year-old anatomical brain features were considered (Shi et al., 2011). Each subject's segmentation was translated to the diffusion space, by registering the T1w image to the diffusion baseline volume using affine registration based on mutual information as implemented by IRTK (Studholme et al., 1999).

#### Network node definition

T1w images were used to obtain the parcellation of each subject brain in regions of interest (ROI), according to the Anatomical Automatic Labeling (AAL) atlas (Tzourio-Mazoyer et al., 2002). This atlas provides a parcellation of the brain based on anatomical criteria. AAL defines 90 cortical regions and 16 cerebellar regions. We merged the 16 cerebellar regions into 3 (right cerebellum, left cerebellum and vermis) to simplify the analysis, obtaining 93 regions considered as the network nodes. The standard AAL atlas was used for parcellation in C6 and C10 cohorts, and the specific 1-year-old version of AAL in Shi et al. (2011) was used for C1 cohort. Parcellation was performed using a consistent block-matching algorithm (Warfield et al., 2002; Tristán-Vega and Arribas, 2007) which provided the elastic transformation matching the atlas template to each subject T1w image.

#### Tractography

Diffusion weighted images (DWI) were processed to estimate the fiber tracts connecting different regions of the brain. For each subject, all DWI were first registered to the baseline in order to correct for eddy current effects and motion artifacts during acquisition (Jenkinson et al., 2012). Diffusion tensor image (DTI) estimation and deterministic tractography were performed using MedINRIA (Toussaint et al., 2007). Fractional anisotropy (FA) threshold was set to 0.1 to ensure the streamlines reaching the GM-WM interface.

#### Connectivity matrix

A pair of brain regions *i* and *j* were considered to be connected if at least one streamline had end-points in the WM-GM interface of both regions, excluding self-loops. Both binary and weighted connectivity matrices were considered. The *(i,j)* element of the binary connectivity matrix was set to one if at least one connection between *i* and *j* existed, and zero if it did not. Regarding the weighted connectomes, at each *(i,j)* matrix element, we defined the connectivity weight as the mean FA value along the streamlines linking the pair of regions *i* and *j*. FA is a measure of the anisotropy of diffusion, indicating the degree of preferred directionality of water diffusion, being influenced by axonal fiber maturation and myelination (Sen and Basser, 2005).

In addition, a normalized version of the FA-weighted connectome was also computed. In order to assess the brain organization independently of the overall network strength^1^, the weighted networks were individually normalized by the total FA weight of all the connections in the network; in such a way that each subject normalized connectome has total network strength equal to 1 (Batalle et al., 2014).

In summary, we defined three connectomes for each subject: binary, weighted by FA (FA-w), and the normalized FA-w connectome (FA-n).

### Brain network analysis

Structural connectomes, obtained as previously described, define brain networks that can be characterized using graph theory metrics, including basic measures, such as the number and weight of the connections, and measures of functional integration and segregation (Rubinov and Sporns, 2010).

Degree and strength are basic measures describing connectivity. For an individual node, the degree is defined as the number of nodes connected to it, while the nodal strength is the sum of the weights of all the connections of this node. In this work, we considered the average degree and strength in the whole network. These metrics can influence the integration and segregation measures, since higher degree/strength indicates more connections at a global level which is generally related to shorter path lengths between nodes.

Integration is the ability to rapidly combine specialized information from the distributed brain regions (Rubinov and Sporns, 2010). Measures of integration are related to the so-called path length, that is, the distance between two nodes. In a binary network, it is quantified according to the minimum number of steps required for moving from a given node to another, whilst in a weighted network it is defined as the weight of all the steps required for moving from a given node to another one. In this study, we considered global efficiency, which is a typical measure of integration inversely related to the path length. High values of global efficiencies are related to short distances between network nodes, allowing for fast communication between pairs of brain regions.

Segregation in the brain is the ability for specialized processing to occur within densely interconnected groups of brain regions (Rubinov and Sporns, 2010). Measures of segregation are related with clustering around individual nodes. Typical measures are the clustering coefficient and the nodal efficiency. Clustering coefficient of a node is a parameter that measures the number of neighbors of this node that are also neighbors of each other. The efficiency of a node is the global efficiency of its associated subnetwork. In this study we considered the average of these metrics in the whole network, that is, average clustering coefficient and average nodal efficiency, also known as local efficiency. High local efficiency and clustering coefficient are related to both a highly segregated network and to a high number (in binary connectomes) or weight (in weighted connectomes) of connections between groups of regions.

### Neurodevelopmental assessment

Neurodevelopmental outcome was assessed for all infants by neuropsychological testing appropriated for each range of age.

Children in the first cohort (C1), scanned at 1 year of age, were evaluated at 20 months of corrected age with the Bayley's Scale for Infant and Toddler Development (BSID-III), which evaluates five different scales: cognitive, language, motor, socio-emotional behavior and adaptive behavior. The scales have scores with a mean of 100 and a standard deviation of 15. Scores lower than 85 were considered as abnormal performance (Anderson et al., 2010). Examinations were performed by a trained psychologist with previous experience in the BSID-III that was not informed about the infant medical history.

Neurodevelopmental outcome of the second cohort (C6) was assessed at 6 years of age by three different tests: executive function was evaluated by the behavior rating inventory of executive function (BRIEF) (Gioia et al., 2000); problematic behavior was assessed using the French version of the strength and difficulties questionnaire (SDQ) (Goodman, 1997, 2001); and cognitive assessment was carried out by the French version of the Kaufman assessment battery for children 1 (K-ABC 1) (Kaufman and Kaufman, 1983).

Behavior rating inventory of executive function (BRIEF) (Gioia et al., 2000) consists of 86 items grouped on 8 clinical scales measuring different aspects of executive functioning in daily life: inhibition, shift, emotional control, initiate, working memory, plan/organize, organization of materials and monitor. In this study, at 6 years of age, we evaluated the behavioral regulation index (BRI), that is the sum of the first three scores; and the metacognition index (MI), defined as the sum of the last five scores. Evaluation consists in a questionnaire completed by the parents, being higher scores associated with poorer executive function.

Strength and difficulties questionnaire (SDQ) (Goodman, 1997, 2001) is a behavioral screening questionnaire for children aged from 4 to 16 years. It includes 25 items grouped on four dimensions that assess problematic behaviors (conduct problems, hyperactivity/inattention, peer problems and emotional symptoms) and a fifth dimension regarding prosocial behavior. Evaluation is performed by a questionnaire completed by parents. In the present study we took into account the hyperactivity/inattention index, since IUGR has been related to a higher risk of these kind of disorders (Baschat, 2011). In this scale, higher scores are associated with more problematic behavior.

K-ABC (Kaufman and Kaufman, 1983) evaluates the children's cognitive ability by means of three scales: the sequential processing (SEQ), simultaneous processing (SIM) and achievement scales. Scores from the SEQ and SIM scales are combined to form a mental processing composite (MPC), which can be interpreted as a measurement of intelligence in the K-ABC. Raw scores are transformed into standard scores with mean 100 and standard deviation 15, with results higher than 85 considered as normal. In our study, SEQ, SIM, and MPC indexes were considered.

Behavioral screening (SDQ) and executive function testing (BRIEF) were also performed at the last cohort (C10), evaluated at 10 years of age. Regarding SDQ, we again focused on the hyperactivity/inattention score. Among BRIEF scales, in addition to the composite BRI and MI scores, the inhibition capacity (INH) score was also considered.

This C10 cohort was also assessed for analogical reasoning abilities using the colored progressive matrices (CPM) test. It provides a global score of the non-verbal intellectual efficiency (Raven et al., 1998). In this study, we considered the raw score, being the higher the score, the better the performance.

Table 1 describes the distribution of ages at which neuropsychological evaluation was performed for each cohort.

### Statistical analysis

Statistical comparisons between IUGR and control groups were performed using general linear models (GLM), including gender and maternal education as cofactors, and GA at birth as a covariate. In the analysis of the case-control differences in network measures, the age at which the MR scan was performed was also included as a covariate, whereas for the case-control comparison of the neuropsychological scores, the age at which the test was performed was considered a covariate. Significance was declared at *p* < 0.05.

Case-control comparisons were performed at each cohort C1, C6, and C10 independently. To describe the longitudinal evolution, a second order polynomial was used to fit the distribution of network metrics in IUGR and control groups along the different ages.

To perform the correlation between neuropsychological scores and network measures, we first corrected the neuropsychological scores by the age at test, and the network measures by the age at MR scan. Afterward partial correlations between the corrected scores and corrected network measures were computed, considering gender, GA at birth, maternal education level and group as confounders.

## Results

### Brain network metrics: developmental trajectories and case-control differences

**Figure 1:**
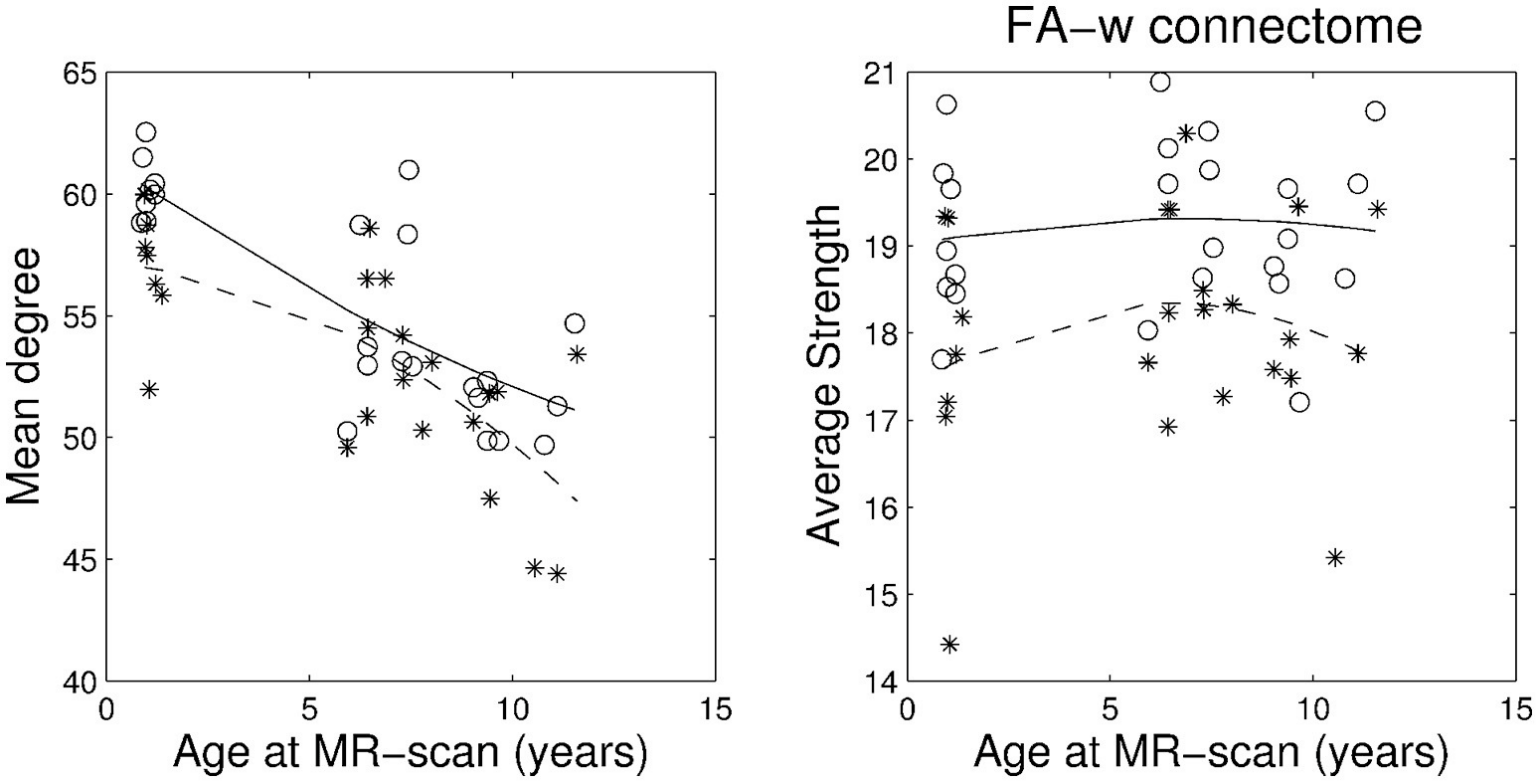
**Basic network metrics: mean degree and average strength in the whole network**. Stars correspond to IUGR children and circles to controls. Solid line, evolution of the metric along age in control group; dashed line, evolution of the metric along age in IUGR group.

**Figure 2:**
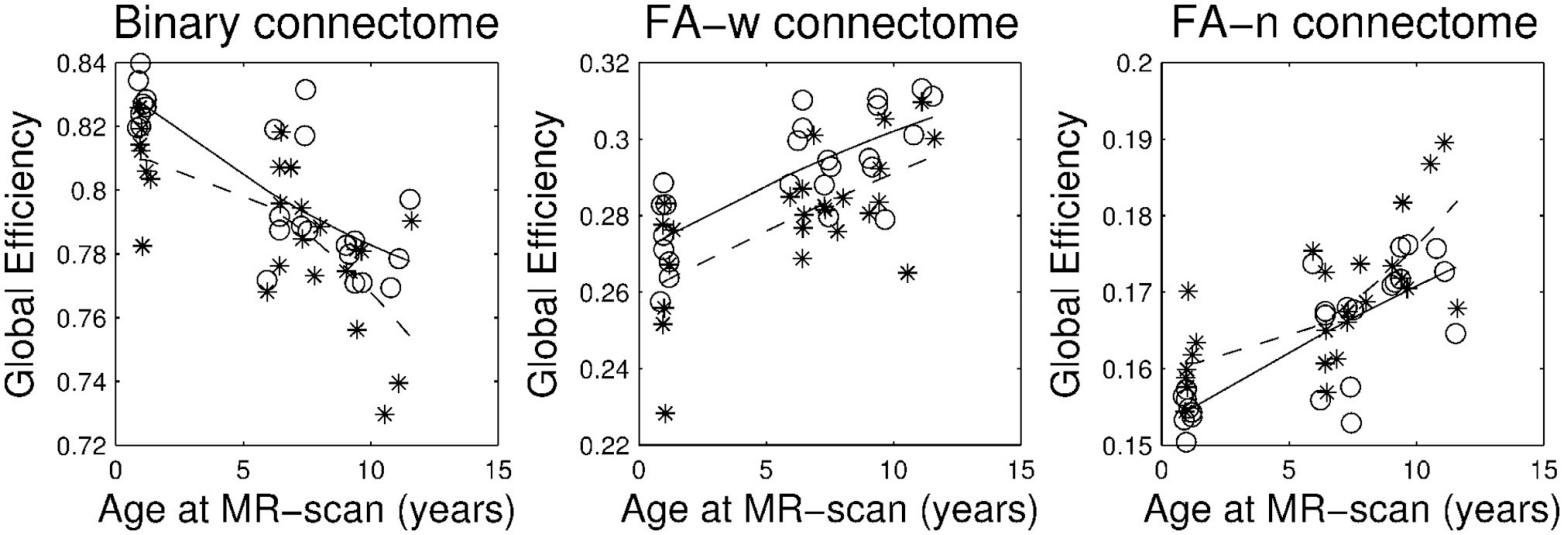
**Brain network integration metric: global efficiency**. Stars correspond to IUGR children and circles to controls. Solid line, evolution of the metric with age in control group; dashed line, evolution of the metric with age in IUGR group.

**Figure 3:**
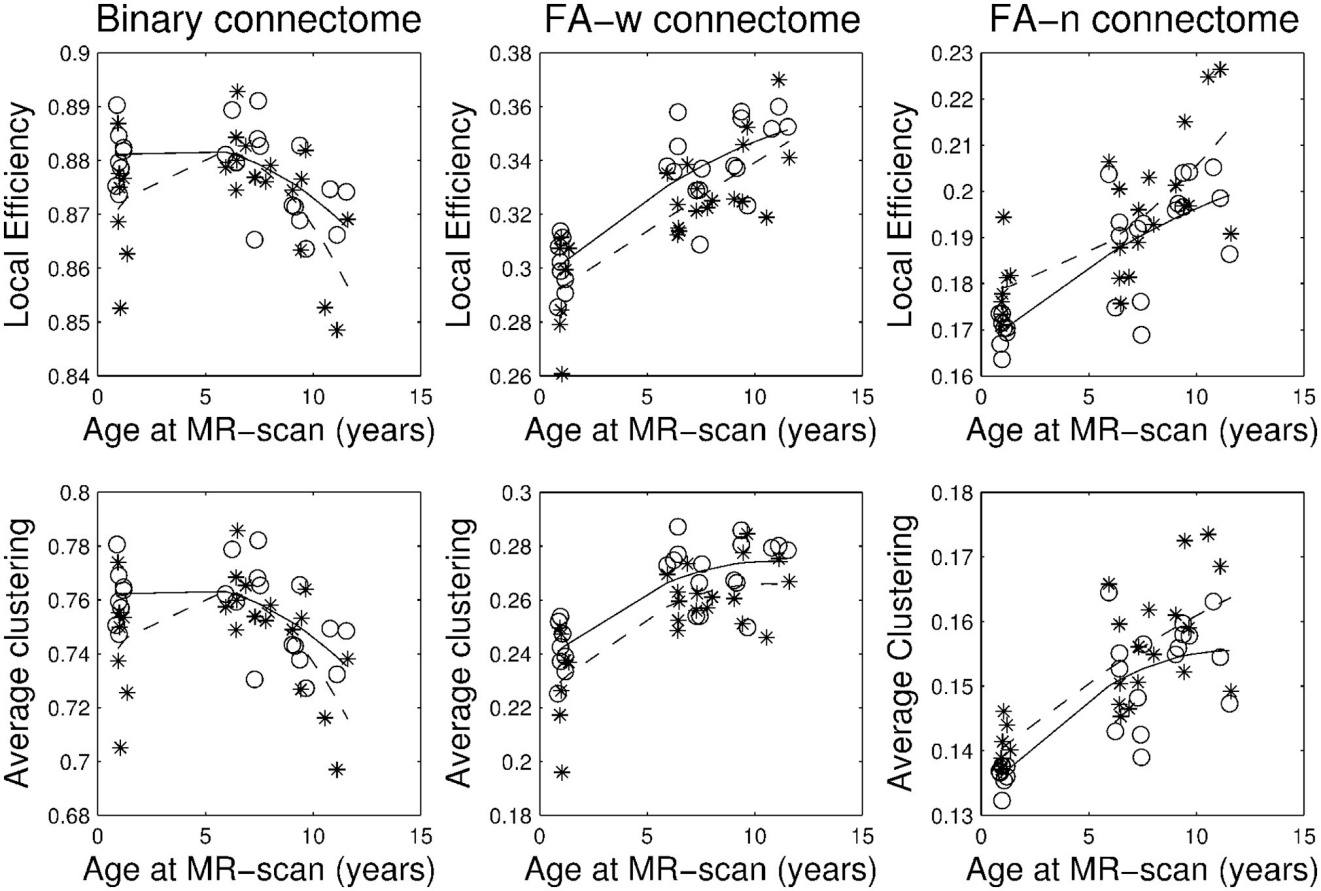
**Brain network segregation metrics: local efficiency and average clustering coefficient**. Stars correspond to IUGR children and circles to controls. Solid line, evolution of the metric along age in control group; dashed line, evolution of the metric along age in IUGR group.

Figures 1–3 show, respectively, the basic (degree or strength), integration (global efficiency) and segregation (local efficiency and clustering coefficient) graph metrics for the three cohorts. Trajectories of these metrics through the different developmental stages for cases and controls were fit by a second order polynomial.

**Figure 4:**
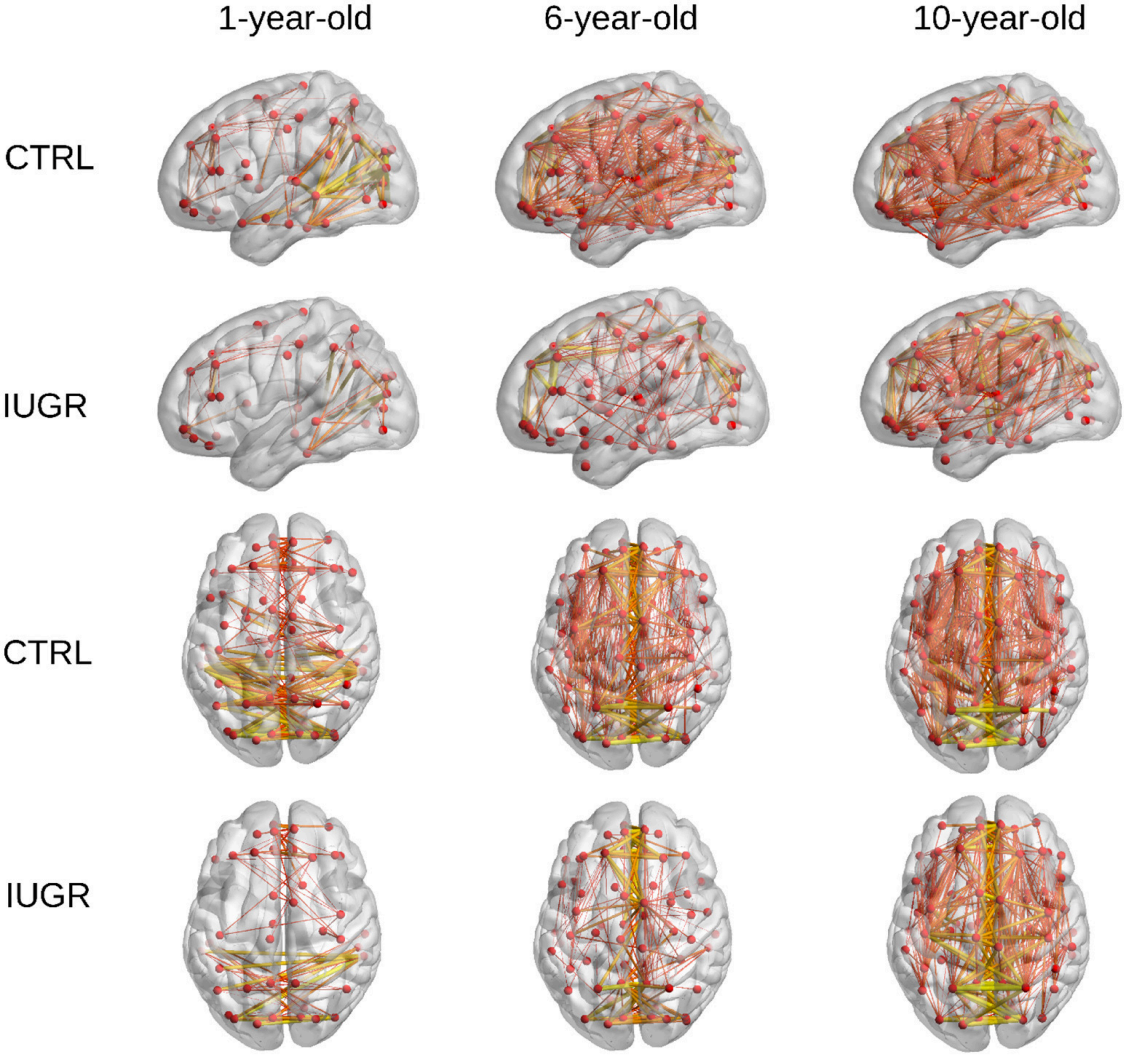
**High-FA connections of the average brain network in control and IUGR children at each range of age**. Only connections with FA weight higher than 0.3 are plotted. Line width and color represents the connection strength.

Furthermore, a representation of the connections with high FA-weight is shown in Figure 4. The average FA-weighted brain network in IUGR and control children at each evaluated time point was computed and connections with an FA-weight higher than 0.3 were depicted in this figure. It can be observed that the number of connections with high FA increases with age, being higher in control children with respect to infants who suffered IUGR at all the evaluated time points.

Table 3 shows the mean and standard deviation of graph metrics for each group. The resulting *p*-value is included for those which were found to be statistically significantly different (*p* < 0.05) between cases and controls or which have shown a statistical trend (*p* < 0.1) to difference. Regarding basic metrics, note that the averaged network degree is the same for all the connectomes, since it does not take into account the weight of each connection. Therefore, we only include this metric in the binary connectome analysis. On the other hand, the connections in the FA-n network were normalized in such a way that the network average strength is the same for all the subjects. Consequently, case-control comparison of FA-n network strength was not performed.

**Table 3:** **Network metrics (mean and standard deviation) in control and IUGR groups at 1, 6, and 10 years of age. ***p***-value of the case-control comparison is only included if ***p*** < 0.1**.

		**1-year-old cohort**			**6-yearl-old cohort**			**10-year-old cohort**		
		**CONTR**	**IUGR**	***p*-value**	**CONTR**	**IUGR**	***p*-value**	**CONTR**	**IUGR**	***p*-value**
BINARY	Degree	60.24 (1.26)	56.87 (2.57)	0.0360	55.13 (3.72)	53.65 (2.96)	n.s	53.14 (1.735)	48.88 (6.522)	n.s
	Global Efficiency	0.827 (0.007)	0.809 (0.014)	0.0370	0.799 (0.021)	0.791 (0.016)	n.s	0.779 (0.009)	0.754 (0.038)	n.s
	Local Efficiency	0.881 (0.005)	0.871 (0.011)	n.s	0.881 (0.008)	0.880 (0.005)	n.s	0.872 (0.006)	0.863 (0.016)	n.s
	Clustering	0.762 (0.011)	0.743 (0.022)	n.s	0.763 (0.016)	0.760 (0.011)	n.s	0.743 (0.012)	0.728 (0.029)	n.s
FA-w	Strength	19.05 (0.93)	17.61 (1.68)	0.091	0.210 (0.010)	0.198 (0.011)	0.026	0.204 (0.011)	0.187 (0.019)	0.0321
	Global Efficiency	0.274 (0.011)	0.263 (0.019)	n.s	0.294 (0.010)	0.282 (0.008)	0.0070	0.301 (0.012)	0.291 (0.015)	0.0213
	Local Efficiency	0.301 (0.010)	0.293 (0.019)	n.s	0.335 (0.014)	0.324 (0.009)	0.054	0.347 (0.013)	0.345 (0.022)	n.s
	Clustering	0.241 (0.010)	0.230 (0.0190)	n.s	0.270 (0.011)	0.260 (0.007)	0.0280	0.273 (0.012)	0.268 (0.014)	n.s
FA-n	Global Efficiency	0.154 (0.002)	0.161 (0.005)	0.0333	0.164 (0.007)	0.167 (0.006)	n.s	0.172 (0.004)	0.183 (0.019)	n.s
	Local Efficiency	0.170 (0.003)	0.179 (0.008)	0.0358	0.186 (0.012)	0.191 (0.010)	n.s	0.198 (0.006)	0.218 (0.032)	n.s
	Clustering	0.136 (0.002)	0.141 (0.003)	0.0194	0.150 (0.009)	0.154 (0.007)	n.s	0.156 (0.005)	0.169 (0.020)	n.s

### Neuropsychological assessment: case-control differences

The between-groups comparison of the cognitive outcome showed that IUGR is related to poorer executive function and higher risk of hyperactivity as evaluated by the neuropsychological tests. Albeit executive function and hyperactivity are better evaluated by tests at school age, a tendency to poorer neurobehavioral outcome was already present at 2 years of age, as assessed by BSID-III. At this age, even though differences in the test outcome were not statistically significant, a non-significant trend to lower scores was observed in cognitive, language, motor, and socio-emotional scales for IUGR children.

Poorer executive function was found in IUGR subjects, associated with higher BRIEF scores at 6 and 10 years of age, with a statistically significant difference at 10 years of age for BRI (*p* = 0.0422) and MI (*p* = 0.0262) scores. In addition, for the 10 year-old cohort a non-significant trend to increased INH scores was also found (*p* = 0.0706).

Higher risk for hyperactivity disorder was also identified in the IUGR group, with an increased SDQ hyperactivity score in IUGR children both at 6 and 10 years of age, statistically significant (*p* = 0.0029) in the C10 cohort.

Tests evaluating more generic cognitive abilities, such as K-ABC at 6 years of age, and CPM at 10 years of age, did not show statistically significant differences, but a tendency (*p* = 0.0980) of IUGR subjects having lower SIM scores, that is, worse performance in simultaneous processing.

### Correlation between neuropsychological scores and brain network metrics

Significant correlations between cognitive outcome and network metrics were found in the three age groups. Neurobehavioral performance at 2 years of age was correlated with brain network structure at 1 year of age, namely cognitive, motor, and socio-emotional scores of the BSID-III were significantly correlated to network metrics, as shown in Table 4.

**Table 4:** **Significant correlations (*p* < 0.05) between network metrics at 1 year of age and BSID-III scores at 2 years of age in C1**.

		**Cognitive**	**Motor**	**Socio-Emotional**
FA-w	Strength	0.6359 (*p* = 0.0435)	n.s	0.9001 (*p* = 3.8e−6)
	Global Eff.	0.6922 (*p* = 0.0187)	0.6842 (*p* = 0.0359)	n.s
	Clust. Coef.	n.s	n.s	0.6997 (*p* = 0.0285)
FA-n	Local Eff.	n.s	n.s	−0.8040 (*p* = 0.0025)
	Clust. Coef.	n.s	n.s	−0.8778 (*p* = 4.2e−5)

Network organization was also correlated to executive function at 6 and 10 years of age. Table 5 shows the significant correlations between BRIEF scores and the network metrics both at 6 and 10 years of age. No significant correlation was found with the MI index at 6 years of age.

**Table 5:** **Significant correlations (***p*** < 0.05) between network metrics at 6 and 10 years of age, and its respective BRIEF scores in the C6 and C10 cohorts**.

		**6-year-old cohort**	**10-year-old cohort**		
		**BRI**	**BRI**	**MI**	**INH**
Binary	Degree	n.s	n.s	n.s	−0.6024 (*p* = 0.0235)
	Global Eff.	n.s	n.s	n.s	−0.6024 (*p* = 0.0023)
	Local Eff.	−0.6182 (*p* = 0.0374)	n.s	−0.5967 (*p* = 0.0256)	−0.7132 (*p* = 0.0023)
	Clust. Coef.	−0.6242 (*p* = 0.0345)	n.s	−0.6165 (*p* = 0.0188)	−0.6821 (*p* = 0.0051)
FA-w	Strength	n.s	n.s	−0.5558 (*p* = 0.0449)	−0.5921 (*p* = 0.0275)
FA-n	Global. Eff.	n.s	0.6015 (*p* = 0.0239)	n.s	0.6737 (*p* = 0.0023)
	Local Eff.	n.s	0.5810 (*p* = 0.0322)	n.s	0.6327 (*p* = 0.0142)

Hyperactivity, as assessed by the SDQ innatention/hyperactivity index, was significantly correlated to the FA-w connectome in the C10 cohort, as shown in Table 6, although no significant correlation was found in C6.

**Table 6:** **Significant correlations (*p* < 0.05) between network metrics at 10 years of age and the SDQ hyperactivity/inattention scores**.

		**10-year-old cohort**
FA-w	Strength	−0.6334 (*p* = 0.0449)
	Global Eff.	−0.9239 (*p* = 3.4e−9)
	Local Eff.	−0.8366 (*p* = 0.0002)
	Clust. Coef.	−0.8310 (*p* = 0.0002)

Non-verbal intellectual efficiency, as measured by CPM score, was correlated to FA-w average strength at 10 years of age (rho = 0.6344, *p* = 0.0138), while no significant correlations were found between the cognitive ability evaluated by general KABC scores and the structural brain network metrics, neither at 6 nor at 10 years of age.

## Discussion

Brain connectivity evolves during development, starting prenatally and continuing into adolescence (Hagmann et al., 2010; Yap et al., 2011; Dennis and Thompson, 2013; Ball et al., 2014; Dubois et al., 2014). Along with this, perinatal conditions, such as IUGR and prematurity have been shown to influence the normal brain development, affecting network connectivity in the short- and long-term (Batalle et al., 2012; Ball et al., 2013; Fischi-Gómez et al., 2015). This altered brain connectivity has been related to the impairment in cognitive and social functions that have been described in childhood and adolescence as a consequence of IUGR (Geva et al., 2006; Wiles et al., 2006; Leitner et al., 2007; Heinonen et al., 2010; Baschat, 2011; Guellec et al., 2011; Levine et al., 2015). In this study, we used connectomics to evaluate brain networks from early infancy to school age in preterm born children with or without IUGR, and to compare the developmental network trajectories in both populations, as well as their relationship to social and cognitive performance.

### Evolution of brain networks

Plots of the network metrics at different ages (Figures 1–3) have shown two different patterns: one associated to the binary network (degree, binary efficiencies, and binary clustering coefficient), decreasing with age; and the other associated to the FA-w/FA-n connectomes, increasing with age.

Interestingly, these two patterns may reflect the two main processes occurring during brain development and maturation: pruning and myelination. Starting in the fetal life, the wiring process of the brain includes neuronal and synaptic overproduction followed by cellular apoptosis, axonal retraction and synaptic pruning to remove redundant connections. This pruning process extends from the end of gestation to childhood (Innocenti and Price, 2005; Stiles and Jernigan, 2010; Dubois et al., 2014). Fiber myelination occurs in parallel with this neural pruning, extending in time beyond late adolescence (Miller et al., 2012). Myelination favors the conduction of the nerve impulse along fibers and has been associated with an increase in FA values, very prominent during the first years of postnatal life (van der Knaap and Valk, 1995; Dubois et al., 2014). From this perspective, it seems logical to hypothesize that the pruning effect could be more related to the binary network metrics, describing which regions are connected to which regions, whilst the myelination process would have a more prominent influence in the FA-w or FA-n networks, that take into account the differences in the mean FA of the connections.

Accordingly, binary network metrics are mainly associated with the structure and organization of the connections themselves, but not with the maturational state of these connections. In this sense, average nodal degree is associated with the number of connected regions and our results showed a decrease with age, what could be viewed as related to the synaptic pruning. Along the same line, efficiency and clustering are also influenced by the number of regions connected and appear to decrease with age. Segregation metrics in the binary connectome follows a different pattern of evolution, showing a very slight increase from 1 to 6 years of age and then a decrease to 10 years. This fact can be explained by a decrease in the number of connections with age (and therefore a decrease in network global efficiency), as measured by the network degree, together with an increase in the organization of the remaining connections in subnetworks, increasing the ability for specialized processing. Previous analyses of longitudinal changes in the binary brain network of healthy subjects have described contradictory findings. For instance, the clustering coefficient was shown to be increased in the early neonatal period by van den Heuvel et al. (2015), while a decrease from birth to 6 months of age followed by a further increase in adulthood was described by Tymofiyeva et al. (2013). The path length (inversely related to global efficiency) has been shown to increase in the early neonatal period and decrease in adulthood by Tymofiyeva et al. (2013), while (van den Heuvel et al., 2015) detected a decrease of path length in the early neonatal period. In the analysis of neonates, 1- and 2-year-old children, Yap et al. (2011) had shown an increase of local efficiency but no differences in global efficiency. An explanation of these different findings can be seen in methodological issues, such as different parcellation schemes, tractography algorithms or different criteria to define connectivity, as well as differences in the cohorts, especially in the analyzed age ranges. In fact, our results showed an inflection point in the evolution of both local efficiency and clustering coefficient during development from 1 to 10 years of age, with an initial increase until 6 years of age and then a decline to 10 years. This change of trajectory can be missed when only neonatal and adulthood periods are considered, or if not enough intermediate time points are evaluated. In addition, regarding our findings, it is interesting to highlight that both IUGR and control subjects follow parallel trajectories along time, with IUGR binary metrics always being lower than control subjects' metrics.

Regarding FA-w measures, which take into account both neural myelination and organization (Sen and Basser, 2005), our results have shown an increase in both integration and segregation metrics with age, while FA strength appears to be constant. This constant FA network strength can be explained by the combination of the decrease in the number of connections and the increase in the FA weight of such connections. As in the case of binary metrics, our results indicated a parallel trajectory between IUGR and healthy subjects, with IUGR infants showing reduced values of network metrics already at 1 year of age. These results are in line with the evolution of FA-w networks described in healthy neonates (from 30 to 40 weeks) by van den Heuvel et al. (2015), showing an increase in clustering coefficients and a decrease in path length (what would be associated with an increase in global efficiency), a tendency that still persists in our cohorts, from 1 to 10 years of age. Connectome analysis considering FA-related weights for the connections has shown similar results. Increase in network strength, global and local efficiency was described in Chen et al. (2013) in a study of subjects from 6 to 29 years of age in a cohort evaluated between 9 and 15 years of age (Koenis et al., 2015).

The increase in FA-w connectome metrics is also reflected in Figure 4, which shows the connections with high FA weights (FA > 0.3) in the average brain network of each group under analysis. Although the binary degree indicated that the total number of connections decreases with age, the connections in early stages have lower FA values, resulting in a smaller number of links whose FA-weight is higher than 0.3. Myelination and maturation occurring with age leads to increased FA values (Oishi et al., 2013; Krogsrud et al., 2016), and results in an increase of the number of high FA weighted connections in the older cohorts.

With regards to the FA-n network metrics, the developmental trajectory was similar to the one described for the FA-w, showing parallel trajectories for IUGR and controls. However, in this case, IUGR showed increased values on their network metrics when compared with controls, as a consequence of the connectome normalization, which will be further discussed in the next section.

Brain connectivity at different age has been assessed in several studies using different criteria to describe or weigh connections: connectivity density (Hagmann et al., 2010); apparent diffusion coefficient (ADC) (Hagmann et al., 2010; Huang et al., 2015); probability of connections (Huang et al., 2015) number of streamlines (Dennis et al., 2013; Zhao et al., 2015) and correlation between GM volumes (Fan et al., 2011). Although the differences in the definition of connectivity do not allow a direct comparison of these results, patterns of increased global efficiency (or decreased path length) throughout development are described in all of them, similar to the pattern we have observed in both IUGR and control children. Regarding the segregation metrics, a decrease in clustering coefficient has been described by several authors (Hagmann et al., 2010; Huang et al., 2015; Zhao et al., 2015), similar to the decrease in clustering measured in our binary network. In these studies, the influence of FA was not considered in the connection weight. When we included FA to weight the connections, the clustering coefficient increased with age as a consequence of the increase of FA with maturation (Oishi et al., 2013; Krogsrud et al., 2016), tapering off in the later period (6–10 years of age), as described in Dennis et al. (2013) for subjects between 12 and 30 years.

### Brain network reorganization in IUGR

Brain network reorganization has already been described in infants and children born with IUGR at 1 and 6 years of age (Batalle et al., 2012; Fischi-Gómez et al., 2015). It has been mainly characterized by a decrease in global and local efficiencies and/or clustering of the FA-w connectome compared to controls. Our results are coherent with these previous findings and show the persistence of these differences in the brain network measures at all three developmental ages, even though these differences appeared more pronounced in the youngest cohort. Changes in network parameters were shown in the three different connectomes we considered: binary, FA-w and FA-n.

Figures 1–3 and Table 3 show a tendency of graph measures from binary connectomes to be decreased in IUGR children. This decrease was found to be statistically significant for binary network degree and global efficiency in the 1 year cohort, but this significance disappears with age. Binary connectome is related to the skeleton of the brain network, that is, it evaluates the existence or absence of connections between regions, disregarding how strong the connections are (Rubinov and Sporns, 2010). Therefore, our results suggest that IUGR leads to a reduction of structural connections between brain regions, especially important in the early developmental period. Differences are less evident later in life, which allows us to hypothesize that in this later period IUGR is mainly associated with a different maturation of the connections rather than with a lack of connections. This idea is supported by the results obtained in the FA-w connectome, sensitive to fiber myelination and/or maturation (Sen and Basser, 2005).

The graph measures associated to FA-w connectome were decreased in IUGR children, as shown in Figures 1–3 and Table 3. Statistically significant differences were present at all three evaluated ages. The FA-w network strength was significantly reduced in IUGR from 1 to 10 years of age. This difference is also illustrated by Figure 4, where lower number of high-FA connections are observed in each average IUGR brain network when compared with their respective average control networks. Differences in FA have been shown to persist even in adulthood, as described by Eikenes et al. (2012) in a 20-year-old cohort. Differences in the connection organization were found at 6 and 10 years of age, significantly decreased global efficiency in both cohorts, and significantly decreased local efficiency and clustering at 6 years of age in IUGR, suggesting that the IUGR brain shows a different brain network organization that persists in childhood. Although we did not find statistical significance in the global and local efficiency of the FA-w connectome at 1 year of age, we observed values that tended to be lower in IUGR infants with respect to controls. In a prior study with a larger cohort (Batalle et al., 2012), IUGR was shown to be associated with a significant decrease of these two network metrics at one year of age. Regarding the 6-year-old cohort, our results using a different methodological approach were coherent with the network metrics shown in Fischi-Gómez et al. (2015).

Finally, the FA-n connectome allows to take into account differences in the network organization regarding the distribution of the connections and their relative individual strength (FA weight), independently of the total network strength (sum of FA in the connections of the entire brain). That is, it can evaluate if the IUGR and control infant brain networks are still different even if they were equal in terms of the average FA of the whole-brain network. Graph measures associated to the normalized connectome show an inverse behavior compared to binary and FA-w network metrics, that is, IUGR values are increased with respect to control values. An increase in the efficiency of the normalized network could be related mainly to two factors: first, a lower number of connections, when the total network strength is set to 1 by normalization, each individual connection has relatively higher weight (FA) than in more connected networks, where the fix total strength must be split into a higher number of connections; and second, a different distribution of the FA-weights of the connections between the different regions in the brain, which has been previously suggested by Batalle et al. (2014) as a potential compensatory mechanism adapting the brain network to the reduced resources. Even though it is difficult to disentangle the individual contribution of these two effects on the increase of FA-n network metrics in IUGR, we can hypothesize that IUGR is related to both a connection decrease and a network re-organization to obtain efficient connectivity from such a limited infrastructure (less organized and/or myelinated axons). Global and local efficiency and clustering coefficient were found to be significantly increased in IUGR cohort, pointing to a reorganization of the brain network in IUGR to cope with the reduced infrastructure and maturation. This connectome normalization approach has been previously used in the analysis of the long-term effects of IUGR in the rabbit model (Batalle et al., 2014) where a significant increase in network metrics was described associated with the normalized generalized fractional anisotropy (GFA)-weighted connectome and a tendency to lower efficiencies in the GFA-weighted connectome in IUGR. This pattern of alterations agrees with our findings in the children cohort. Albeit absence of significance, the increase of FA-n network metrics is still present in the older cohorts.

### Correlation between network metrics and neuropsychological scores

As social and cognitive problems have been previously described in children with IUGR, together with general neurodevelopmental evaluations (BSID-III, K-ABC, and RAVEN), neuropsychological assessment specifically focused on these impairments were administered in form of specific questionnaires (SDQ hyperactivity/inattention score and BRIEF). Results showed that IUGR children performed worse in almost all the scores when compared to controls. The differences were statistically significant in the measures of hyperactivity/inattention and executive function (SDQ and BRIEF) at 10 years of age, which supports the idea of a specific pattern of neurodevelopmental impairments in IUGR children, related to social cognitive problems. Executive dysfunction is more easily detectable in older children, which could explain significant differences appearing only in the older cohort. These findings are coherent with previous studies showing higher hyperactivity and conduct problems associated with IUGR at school age (Wiles et al., 2006).

Since structural and functional maturation of neuronal pathways connecting individual brain regions have been directly related with the successful development of cognitive motor and sensory functions (Paus, 2010), changes in brain structure associated with IUGR may lead to impairments in the neurobehavioral outcome of these children. Consequently, different measures computed from brain MRI have been shown to correlate with neurodevelopmental performance in our cohorts.

Changes in connectivity of the neonatal brain have been correlated to BSID-III scores in studies with IUGR and prematurely born children (Batalle et al., 2012; Ball et al., 2015). It has also been correlated to more severe disorders, such as cerebral palsy, where an increase in brain connectivity was related to improved functional abilities (Englander et al., 2015) in a cohort of children between 1 and 5 years of age.

In agreement with these previous findings, our study shows correlation between the cognitive, motor and socio-emotional scores in BSID-III assessed at 2 years of age and the network metrics evaluated at 1 year of age. Coherently with the fact that metrics computed from the FA-w connectome were shown to be decreased in IUGR and FA-n network metrics were increased in IUGR, positive correlation was shown between FA-w metrics and neurodevelopment outcome, while a negative correlation was found between FA-n metrics and BSID-III scores. It is interesting to note that while cognitive and motor scores are correlated with an integration metric (global efficiency), socio-emotional scores are related to segregation metrics (local efficiency and clustering), pointing to a difference in processing of these brain functions. Motor function and also general complex cognitive processing requires efficiency in long range connectivity, for instance, the cortico-basal-ganglia-thalamo-cortical loops, whereas the socioemotional functioning has been associated with specific short cortico-cortical networks (Fischi-Gómez et al., 2015). Therefore, this relationship with integration and segregation metrics, respectively, could be related with the fact that motor function has been associated to projectional long-range connectivity, while socio-emotional function has been more associated to cortico-cortical networks and association fibers.

In the 6-year-old cohort, correlation was found to be significant between the binary segregation metrics and BRI index, which describes behavioral regulation, being composed of inhibition, shift and emotional control scores. Although not reaching statistical significance, differences were found at this age between IUGR and control, higher BRI values (higher risk of disorder) were obtained by IUGR children, and this score was negatively correlated with the network metrics. Binary segregation metrics are related to the number of regions that are interconnected, forming clustering of specialized processing, without taking into account the strength of the connections.

Finally, the brain network metrics in the 10-years-old cohort were strongly correlated with both BRI and SDQ hyperactivity/inattention score. In this case, correlation is significant both in integration and segregation metrics of the binary, FA-w and FA-n. The higher correlations were found between the network metrics and the SDQ hyperactivity/inattention score (correlation coefficients higher than 0.8 for FA-w global and local efficiency and clustering coefficient), showing a clear relationship between the reorganization of brain connectivity and these attention impairments. Inhibition capacity measures (INH) were similarly correlated with network metrics and associated with the binary, FA-weighted and FA-normalized connectomes. Note that the sign of the correlation relates higher risk (higher SDQ or INH scores) with brain network organization associated with IUGR (lower binary and FA-w network metrics, and higher FA-n network metrics).

An association between structural brain changes and attention deficit-hyperactivity disorder (ADHD) has already been described in previous studies, including changes in FA of specific fiber tracts (de Zeeuw et al., 2012) or specific regions (Kobel et al., 2010), as well as alterations in functional connectivity (Wang et al., 2009). Along with this, previous studies have indicated higher hyperactivity and conduct problems associated with IUGR at school age (Wiles et al., 2006), describing alterations in executive function, attention and memory persisting at 20 years of age (Østgård et al., 2014b). Therefore, our study corroborates the higher risk for IUGR children to have brain network abnormalities disposing them for hyperactivity/inattention disorders in childhood.

### Strengths and limitations

This study presents an evaluation of brain networks from infancy to late childhood, taking into account structural alterations associated with IUGR. Several studies have already shown the evolution of brain networks during development (Hagmann et al., 2010; Dennis and Thompson, 2013; Huang et al., 2015), but the influence of adverse perinatal condition was not considered in these analyses. Although structural changes at some given neurodevelopmental period have been previously described, the changes in brain networks from infancy to early adolescence have not been reported. Actually, longitudinal analyses of IUGR have mainly focused on the neuropsychological outcomes (Leitner et al., 2000, 2007; Geva et al., 2006), but correlation between these outcomes and changes in brain structure was not described in these studies. One of the strengths of our study is the fact of having very well characterized cohorts with both image acquisitions and neuropsychological evaluation available so both cognitive and structural differences, and the relationship between them, could be assessed. From a structural point of view, long-term brain reorganization associated with IUGR had been described in Fischi-Gómez et al. (2015) at 6 years of age, but, to the best of our knowledge, this is the first study that analyzes brain networks in IUGR children from early infancy up to 10 years of age. To describe the brain network organization along time, this analysis combines different cohorts from Hospital Clìnic de Barcelona and Hospitaux Universitaires de Gèneve that have been acquired with similar imaging parameters. This allows us to have MR-scans of children at 1, 6, and 10 years of age, although we could not have the same subjects in the three time points. This is a common problem to most of the studies describing evolution of brain networks along development (Dennis and Thompson, 2013; Hagmann et al., 2010; Huang et al., 2015). Nevertheless, it deserves to be highlighted that some of the subjects in the 6 and 10 year-old cohorts were the same, leading to stronger reliability in the longitudinal comparison.

The availability of a variety of neuropsychological test evaluating the children performance at every range of age, and the combination of these results with the brain structural measurements provide new insights in the description of the short- and long-term effects of IUGR. However, we acknowledge that the sample size at every age group is relatively small. But even with this small sample size, a similar pattern of differences between IUGR and controls was observed at each time point. Small sample size could explain the lack of significant differences in some measures, both in neuropsychological scores and network metrics.

The acquisition parameters are slightly different in C1 compared to the other two cohorts, mainly the voxel size, which for obvious reasons is smaller in the C1 cohort. It is related with the difference in brain size between 1-year-old children and older subjects, which requires to have smaller voxels to have sufficient image resolution. For this reason, in the analysis of connectivity we have considered the FA weighted networks, since it is a relative parameter (Westin et al., 2002), which minimizes the possible influence of differences in voxel and brain size between cohorts. Only global or average local metrics have been considered in the study. It would be interesting to identify regional alterations and patterns of network nodes or specific connections showing differences between IUGR and controls. However, due to the small sample size and the high number of nodes (93) and connections (around 8000) this would have proven statistically difficult.

## Conclusions

The development of brain networks from infancy to childhood is characterized by an increase in the efficiency and clustering of the FA-related connectomes (both FA-weighted and FA-normalized), and a tendency to decreased efficiency and clustering coefficients of the binary connectome, especially in the later infancy period. These changes can be associated, respectively, with an increase in structured and myelinated connections along with a pruning of less organized connections. The longitudinal trajectory is similar in both IUGR and control preterm born children, although a delay could be observed in IUGR with respect to control children, related to a pattern of structural alterations in brain network associated with this condition that persists, but lessens with age. Namely, a decrease in the FA weighted metrics and increase in normalized FA networks was found, which is likely related to lower neural myelination and/or organization in IUGR children. These structural differences are related to a higher risk of social cognitive disorders in IUGR, being especially relevant the association with executive dysfunction and hyperactivity/inattention behavior.

## Author contributions

EM, EF, PH, JT, and EG contributed to the conception and design of the work. EE, EG, CB, and PH were in charge of the subject recruitment and follow-up, cohort characterization, neuropsychological tests and image acquisitions. EM, EF, DB, JT were focused in the connectomics analysis, development of algorithms to estimate and analyse brain networks. EM, EF, and PH performed the analysis and interpretation of the results and draft the work. All the authors revised and approved the final manuscript.

### Conflict of interest statement

The authors declare that the research was conducted in the absence of any commercial or financial relationships that could be construed as a potential conflict of interest.

## Footnotes

^1^In a weighted network, the strength of a node (nodal strength) is the sum of the weight of all the connections of the node. Network strength is defined as the sum of the nodal strength of all the nodes in the network. In our case, the connection weight is the average FA along the connection.
