# Corrigendum: Structural Brain Network Reorganization and Social Cognition Related to Adverse Perinatal Condition from Infancy to Early Adolescence

**DOI:** 10.3389/fnins.2017.00257

**Published:** 2017-05-09

**Authors:** Emma Muñoz-Moreno, Elda Fischi-Gomez, Dafnis Batalle, Cristina Borradori-Tolsa, Elisenda Eixarch, Jean-Philippe Thiran, Eduard Gratacós, Petra S. Hüppi

**Affiliations:** ^1^Fetal i+D, Fetal Medicine Research Center, Barcelona Center for Maternal-Fetal and Neonatal Medicine (Hospital Clínic and Hospital Sant Joan de Deu), Institut d'Investigacions Biomèdiques August Pi I Sunyer, University of BarcelonaBarcelona, Spain; ^2^Experimental 7T MRI Unit, Institut d'Investigacions Biomèdiques August Pi I SunyerBarcelona, Spain; ^3^Signal Processing Laboratory 5, École Polytechnique Fédérale de LausanneLausanne, Switzerland; ^4^Division of Development and Growth, Department of Pediatrics, University Hospital of GenevaGeneva, Switzerland; ^5^Centre for the Developing Brain, Division of Imaging Sciences and Biomedical Engineering, King's College LondonLondon, UK; ^6^Centre for Biomedical Research on Rare DiseasesBarcelona, Spain; ^7^Department of Radiology, University Hospital Center and University of LausanneLausanne, Switzerland

**Keywords:** connectome, intrauterine growth retardation, birth weight, executive function, neurodevelopment, preterm infants

In the original article, we omitted a reference to Réveillon et al. (2016) regarding the description of the neuropsychological tests performed by the children and the association between IUGR and hyperactivity/inattention symptoms. This reference is cited in the description of the third cohort (Section *Materials and Methods. Subjects*) and in the *Correlation between Network Metrics and Neuropsychological Score* section, as appeared below. We also had neglected to thank the invaluable contribution of the team involved in recruitment, imaging acquisition, and neuropsychological testing. The revised version of the acknowledgments is provided below. The authors apologize for the oversight. These errors do not change the scientific conclusions of the article in any way.

## Materials and methods

### Subjects

• Third cohort (C10) was composed of 16 subjects, recruited at the Hôpitaux Universitaires de Genève (HUG). Both MR imaging and neurobehavioral assessment was performed at 10 years of age (Réveillon et al., 2016).

## Discussion

### Correlation between network metrics and neuropsychological scores

…These findings are consistent with previous studies showing higher hyperactivity and conduct problems associated with IUGR at school age (Wiles et al., 2006; Réveillon et al., 2016).

### Conflict of interest statement

The authors declare that the research was conducted in the absence of any commercial or financial relationships that could be construed as a potential conflict of interest.
